# Effective interactive engagement strategies for MOOC forum discussion: A self-efficacy perspective

**DOI:** 10.1371/journal.pone.0293668

**Published:** 2023-11-09

**Authors:** Wei Wei, Jia Liu, Xiaoshu Xu, Kimberly Kolletar-Zhu, Yunfeng Zhang

**Affiliations:** 1 Faculty of Applied Sciences, Macao Polytechnic University, Macao, China; 2 Zunyi Medical University Zhuhai Campus, Zhuhai, China; 3 School of Foreign Studies, Wenzhou University, Wenzhou, China; 4 Faculty of Languages and Translation, Macao Polytechnic University, Macao, China; Universite Amar Telidji Laghouat, ALGERIA

## Abstract

This mixed methods sequential explanatory study identified and explained the features of engagement strategies for MOOC forum discussion that help low-achieving students make significant progress. Triangulated data were collected from MOOC learners’ (n = 335) scores in two reading assessments, their posts to the embedded online discussion forum, and their self-reflection learning journals. Based on learning progress between pre- and post-assessment tasks, MOOC learners are divided into three groups: 1) little, 2) moderate, and 3) significant progress. According to a statistical analysis of the quantified posts, surprisingly, the low-scoring students from the pre-test who demonstrated significant progress later engaged in significantly fewer peer-peer and peer-teacher interactions in the online discussion forum. Guided by self-efficacy literature, the reflective journals of these learners suggested that 1) learner-content interactions may help them advance learning and obtain new information and linguistic knowledge from the peer-made learning materials in the discussion forum; 2) they did not share and exchange ideas and answers with their peers. Instead, they prefer learning from others’ discussions and wish to get quick feedback and suggestions on their contributions to the discussion forum; and 3) peer-peer and peer-teacher interactions were proposed as two solutions to regulate their online learning experience as they lack self-discipline and time-management skills. Implications include teachers’ continuous support to encourage low-achieving students to learn peer-generated content and quick feedback on their contributions to the discussion forum.

## Introduction

Engaging with peers, teachers, and materials in online discussion forums has been proposed as a pedagogical solution in MOOCs to promote better learning gains, increase motivation levels, reduce the dropout rate, and increase the completion rate. Some research gaps are validated. First of all, learning progress is rarely mentioned as one of the indicators of academic success in MOOC studies. By using cognitive and behavioral indicators [1–3], most of the previous studies evaluated the effectiveness of MOOC learners’ participation in the online discussion forum with indicators such as dropout rate [4], completion rate [5], grades, and scores on the final exam [6]. Some have attempted to predict MOOC learners’ achievement using behavioral indicators such as the number of comments and replies [5], the number of likes [7], and downloads [6]. Second, linking outcomes to MOOC learners’ behaviors may have some methodological flaws because the actual contributions of participating in online discussion forums can be difficult to measure without a baseline study [8, 9]. Third, its positive contributions to learning outcomes [10] have attracted increased attention from researchers, with more studies currently being conducted in the fields of sciences, economics, and business-related subjects than in the arts and humanities, such as biology [11], medicine [12], statistics [13], and business strategy [7]. Its implications and interactions with language learners have not been thoroughly researched.

Thus, the study bridges the gap by investigating language learners’ engagement with online discussion forums, particularly the strategies and engagement patterns of students with low self-efficacy who make significant progress on the MOOC platform. As addressed in the UN Sustainable Development Goals, education needs to reduce inequalities, and the design, establishment, maintenance, and engagement with digital learning resources is one of the key strategies. Therefore, this investigation and development of effective interactive engagement strategies for low-achieving online learners can make MOOCs a more accessible and sustainable learning platform. The investigation and development of effective interactive engagement strategies for low-achieving online learners can make MOOC a more accessible and sustainable learning platform.

## Literature review

### Engagement and learning outcomes in MOOCs

Previous research has linked specific types of learning engagement in online discussions to either improved or satisfactory learning outcomes. Two types of factors have been identified as facilitators for effective online discussion forums in MOOCs. The first category of facilitating factors pertains to specific interactions and patterns of engagement with posts in online discussion forums [5, 12, 14]. More specifically, interaction and engagement patterns encompass a wide range of behaviors, such as a higher frequency of learner-learner interactions, a greater diversity of learners’ profiles in discussion forums, and more contributions in terms of writing posts or commenting on others’ posts. Interestingly, the frequency of instructor-learner interactions was not found to be a significant predictor of academic success in contrast to the classroom setting. For example, in a study by Elizondo-Garcia and Gallardo [15] on an Energy-Saving MOOC, learner-learner interaction activities such as viewing peers’ comments and contributing to the discussion forum were shown to improve learning outcomes on the subject. Moreover, the diversity of learners’ profiles, including their various levels of expertise, inspired learners to think about problems from various perspectives, which can be achieved by reviewing others’ assignments. Additionally, feedback on others’ posts, such as giving points and compliments, was found to positively correlate with completion status, according to Cohen et al.’s [5] research on a MOOC about the Modern Middle East in 2019. Finally, the number of contributions made by learners strongly predicted a higher completion rate, as demonstrated in Bonafini et al.’s [16] survey of a MOOC titled "Creativity, Innovation, and Change," where students who made more posts and watched more videos achieved higher grades.

The second type of facilitating factor refers to content characteristics such as post-emotions and relevance to the teaching objectives of MOOCs [4, 17]. Xing, Tang, and Pei [4], for example, discovered that students’ exposure to deactivating emotions, particularly positive deactivating emotions, is related to their completion. Deactivating emotions encompasses both positive and negative ones. Learners who experience positive deactivating emotions, such as relaxation, have a positive attitude toward learning activities and outcomes, but they do not devote much time or effort to studying. Conversely, learners who experience negative deactivating emotions, such as hopelessness, have negative attitudes toward learning activities and outcomes, and they make no effort to achieve them. In another study, Wise and Cui [17] compared the degree of association of two types of discussions on the MOOC StatMed’14 (a statistics MOOC) with final grades: discussions related and irrelevant to the course content.

Research has concluded that course content-related discussions promote the development of in-depth conversations involving a broader range of topics and a higher number of contributing posts, which are indicative of students’ learning outcomes. In addition to facilitating factors, Xing, Tang, and Pei’s [4] study discovered that MOOC learners’ exposure to negative activating emotions increases the likelihood of dropout. Negative emotions, including anger, anxiety, and shame, typically result in students devoting all of their time and energy to learning activities but harboring negative opinions about the activities and outcomes.

### Patterns of engagement and learning behaviors

This study identified two distinct approaches to engagement and interaction patterns in online discussion forums: collaborative and independent. Collaborative engagement involves interactions among learners, as well as interactions between learners and instructors. Collaboration in discussion forums, such as posting, liking, replying, commenting, and providing peer feedback on assignments, had a significant positive relationship with students’ study outcomes or performance. Learner-learner and learner-instructor interactions had positive effects on occasion, promoting learner-content interactions; greater participation in discussion forums may indicate greater lecture and assignment coverage [18]. Interestingly, Zhang, Allon, and Van Mieghem [18] found that both large and small group discussions can promote learner-content interaction, as demonstrated by increased quiz completion rates. However, only small group discussions were found to be beneficial to participants’ quiz scores in MOOCs. In contrast, independent modes of engagement, for example, suggested a learner-content pattern, such as interactions with the curriculum [17], viewing instructional videos, and completing quizzes and assignments alone, which had fewer facilitative effects.

When addressing the importance of instructor-learner interaction in MOOC discussion forums, Almatrafi, Johri, and Rangwala’s [12] study went further to identify those posts that require immediate responses from instructors by analyzing their linguistic features. They used the Linguistic Inquiry and Word Count (LIWC) application to analyse posts in a MOOC discussion forum. Each post was divided into 94 features that assessed the social and psychological significance of words. Words like "please" indicate a polite message, whereas "essay" or "quiz" indicate a course-related message.

### Patterns of engagement and learner self-efficacy

Language learners’ self-efficacy, or self-reported confidence level, in engaging with online learning materials and activities has received increasing attention and is perceived as one of the facilitating factors in promoting better learning outcomes for language learners [19]. For example, studies have found that learners who report higher levels of self-efficacy in online language learning are more likely to participate in online discussion forums, complete assignments, and perform better in language assessments [19, 20]. Moreover, learners’ perceived usefulness was influenced by their self-efficacy, which then, indirectly, increased their satisfaction with wireless internet service for academic purposes [21].

Self-efficacy denotes students’ capacity and determination to accomplish a specific task utilizing computer skills [22]. Additionally, it alludes to learners’ overall self-assurance in operating internet functions or applications in a computer-based learning milieu [20]. Self-efficacy has been implemented to account for attitudes toward MOOCs, which is one of the most prominent factors influencing their adoption [22]. According to Alberth [23], three sub-components of self-efficacy concerning academic writing are as follows: 1) ideation; 2) conventions; and 3) self-regulation. Self-efficacy in ideation pertains to writers’ confidence in generating content for written assignments (e.g., ideas, reasoning, and evidence). Conventions correspond to writers’ confidence in articulating their ideas through linguistic skills (e.g., translating concepts into proper discourse structures and written language). Self-regulation involves both self-management and effective control strategies, such as being self-motivated when faced with a challenge and actively seeking support from others during the writing process [24].

Numerous remedies have been proposed to enhance the engagement of low-efficacy EFL students with online learning materials and activities. For instance, Zhang and Cui [25] conducted a survey on remote adult EFL learners from the School of Continuing Education, finding that their motivation and anxiety levels in an autonomous learning environment were associated with their distance learning experience. Additionally, Selcuk, Jones, and Vonkova [26] ascertained that high school EFL learners’ self-confidence and motivation towards writing in English can be improved by receiving compliments and motivational phrases from senior members and leaders in their groups. However, some researchers are doubtful about the correlation between increased self-efficacy and actual progress, especially among language learners with low proficiency and who are accustomed to teacher-centered environments. For instance, Lin [27] conducted interviews with a group of adult EFL learners in Taiwan about their blogging experiences and found that learners’ positive attitudes towards increased self-efficacy in English writing did not result in an increase in blogging activity.

### Research gaps and research questions

The current understanding of online discussion forums in MOOC courses suggests that while content-oriented taxonomies have been developed to analyze the posts and replies in MOOC discussion forums, it is still uncommon to focus on the content and functions of the posts generated by online learners [14, 28]. However, posts and comments on discussion forums are critical indicators of learning outcomes in a language MOOC program [29, 30]. Early intervention for low-motivation and low-engagement MOOC learners is believed to be an effective way to increase completion rates and decrease dropout rates. It is hypothetically suggested that more frequent human interaction between learners and teachers in a MOOC learning environment may promote learners’ engagement level and learning outcomes.

Furthermore, it is still rare to link the types and frequency of content to learners’ academic achievement and self-efficacy level, particularly their progress during their online learning experience. The concept of self-efficacy has been examined in MOOC studies. Most studies identified self-efficacy as one of the significant predictors or moderators for learning outcomes in a MOOC environment, such as academic success [31], completion rate [32], perceived effectiveness of MOOC [33], MOOC learners’ learning persistence [34], and increased motivation level [35]. Some other studies investigated the sources of MOOC learners’ self-efficacy [36]. Last, given the importance of self-efficacy in self-directed learning, other studies examine the extent to which specific pedagogical activities may improve MOOC learners’ self-efficacy, such as co-design and delivery of MOOCs [37, 38]. This study adopts self-efficacy as a framework to understand low-performing students’ strategies in interacting with their peers, instructors, and materials published in MOOC discussion forums. With these two points in mind, this study aims to address two research questions:

1. To what extent can the function of posts be used to explain online learners’ progress during a language MOOC course?
2. Do certain functions positively or negatively contribute to learners’ progress and why?

## Research method

### Research context

The IELTS MOOC course under study was a one-semester selective language course catering to sophomores of various majors at Wenzhou University in China. The International English Language Testing System (IELTS), which is managed jointly by the British Council, IDP Australia, and Cambridge Assessment English, was the primary focus of this course. The project team included five members, with one teacher and one teacher assistant responsible for course instruction and MOOC IELTS materials preparation, respectively. The remaining three members were ICT engineers responsible for the development and management of the MOOC platform. Upon completion of the course offered by the School of Foreign Studies, participants were awarded three academic credits. The primary objective of the IELTS MOOC course was to enhance learners’ academic English proficiency, particularly for those preparing for future studies abroad while simultaneously preparing for the IELTS exam. The course material consisted of various online open resources, including e-books, videos, and self-developed quizzes. The corresponding author of this study received ethics approval from the department research committee, and the participants were informed of the study’s objectives two weeks before commencing the MOOC course through E-mail. Only those students who provided written informed consent were included in the study and were free to withdraw at any time. Pseudonyms were used to protect participants’ identities during the data-coding process.

### Data collection

The present study collected data on learners’ scores in two IELTS reading assessments administered before and after the course, as well as their posts and replies in the discussion forum and their self-reflective journals. The data collection started on February 27, 2019-June 30, 2022, involving four cohorts of students. The score data pertains to MOOC students’ IELTS reading test scores, which were obtained through the use of authentic IELTS Academic reading tests that were adapted to ensure the validity and reliability of the assessment tasks. Each reading assessment was one hour long and consisted of 40 questions, with a maximum score of nine. The texts were approximately 700 words long and covered a range of topics related to ecology, social issues, technology, and culture. The scores were graded on a scale of nine using the IELTS recommended grading scheme, making them comparable and enabling the difference between the pre-and post-test scores to be used as a measure of progress in English academic reading skills.

The MOOC participants’ posts and replies in the discussion forum were obtained in Excel format from the platform, containing the authors’ names, posting times, actual words, and emojis in the posts. To collect the reflective journals, MOOC participants were asked to respond to one guided question: "What are the challenges and difficulties for you to learn English in this MOOC?" The journals could be written in Chinese or English with no word limit, and there were no credits associated with the task. Learners were encouraged to write reflective journals three times during the course: in the third, seventh, and eleventh weeks. Initially, three cohorts of students (n = 367 students) agreed to participate in the study. All were second-year business students majoring in Marketing, Management, Accounting, Logistics and Finance, with an approximate language proficiency level of B2 and an average age of 20, having more than ten years of face-to-face English learning experience. Out of the enrolled students, 335 participants, consisting of 190 males and 145 females, provided their pre- and post-reading test scores, submitted reflective journals three times during the 15-week MOOC, and were granted permission to download and analyze their discussion forum posts.

### Data analysis

A series of ANOVA tests were conducted to assess the differences among the three groups, characterized by their level of progress (little, moderate, and significant) in terms of their contributions to the discussion forums from six perspectives. To assess progress, the difference between participants’ scores on IELTS reading assessment tasks taken before and after the course was calculated. Participants were divided into three groups based on the increase in their scores: those with little progress (n = 84; score increase = 0 or 0.5), those with moderate progress (n = 142; score increase = 1 or 1.5), and those with significant progress (n = 109; score increase >1.5). To measure and compare MOOC learners’ posts on the discussion forum from these three groups, a framework was adapted from Fu, van Aalst, and Chan’s [49] study on the classification of learners’ online asynchronous discussions. The framework consists of six categories (see Table 1), including social and affective, information, questions, ideas, agency, and community-building: meta-discourse. Each post was assigned no more than two codes. The first 60 posts were independently coded by three researchers, and any discrepancies or disagreements were resolved through discussion. To improve consistency, the following coding rules were established:

**Table 1:** Discourse features.

	Labels	Rules
1	Social, affective, communal	Comments with no structure markers/bullet points, emotions
2	Information	Share information and content from other resources: references/news/statistics/notes/quotations
3	Question	Self-questioning, ask questions to teachers, answer and respond to teachers’ questions
4	Idea	Express ideas and respond to teachers’ questions and tasks with structural markers: firstly/secondly
5	Agency	Evaluate/question others’ points with clear judgments
6	Community: meta-discourse	Summarize comments/points from other peers and teachers

The second code, "information," pertains to MOOC participants’ responses and comments that contain references to external sources such as news, textbooks, or reports. It is essential to differentiate between original thoughts and opinions from external sources. Furthermore, the code "questions" should include rhetorical questions and be accurately labeled as such in a post. Lastly, the third code, "ideas," should include logical transition markers such as "in addition," "meanwhile," and "on the other hand" to demonstrate how MOOC learners organize and present their ideas.

For the second research question, reflective journal data from the third group, which made significant progress between the two reading assessments, were analyzed and coded according to self-efficacy literature guidelines (see Table 2). Self-efficacy in this study is defined as learners’ capacity to: 1) generate and learn new ideas from tasks and instructional materials in online discussion forums; 2) share, convey, and communicate with other MOOC learners in the platform; and 3) self-regulate and manage the entire learning process on the platform.

**Table 2:** Codes for reflective journals.

Difficulties and challenges in	Peer—teacher interactions	Peer—peer interactions	Peer—content interactions
Generating and learning new ideas, concepts, and knowledge			
Sharing, conveying and communicate the new knowledge with others			
Self-regulating or managing the whole learning process			

Three researchers interactively coded the data. The first step involved categorizing the reported challenges into three types of engagement and interactions: peer-teacher, peer-peer, and peer-content. In the second step, the researchers employed self-efficacy theory to make informed decisions on the nature of the reported challenges. Any new theme that emerged was subjected to extensive discussions among the researchers. Disagreements, if any, were resolved through consultations with English teachers on the platform, who maintained weekly communication with the MOOC students.

## Results

Table 3 reveals the prevalence of discourse functions in students’ posts, irrespective of their reading assessment scores. Social and ideas emerged as the most frequently employed discourse types, with agency and community being the least frequently utilized. This indicates that students predominantly utilized the online discussion forum to establish rapport and camaraderie, convey their sentiments via peers’ posts, respond to tasks and topics set forth by their instructors, and interact with their colleagues’ contributions by applying the knowledge garnered from lectures, recommended reading materials, and academic literature. Although online forums may not be primarily utilized for questioning and answering purposes, they facilitate the critical assessment and evaluation of the quality of posts shared by fellow students, thus enabling collaborative knowledge construction.

**Table 3:** Descriptive data.

	N	Mean	SD	Skewness
Social, affective, communal	335	11.56	5.10	0.82
Idea	335	5.98	3.20	0.68
Information	335	5.27	2.68	0.95
Question	335	1.66	1.38	1.13
Agency	335	0.06	0.23	3.73
Community: meta-discourse	335	0.04	0.20	4.42

The participants were categorized into three groups, based on the difference in their scores between pre- and post-reading assessments: little, moderate, and significant progress. Table 4 illustrates the score differences among the groups. The group with “significant progress” reported a much lower score in pre-reading assessment (M = 4.742) in comparison with the groups who achieved little (M = 6.238) and moderate progress (M = 5.366). In other words, the interactive strategy pattern reported by the learners in “significant progress” group may represent the highly motivated low-achieving online learners. Results from the ANOVA test (see Table 5) indicate that in comparison with students who achieved little and moderate progress, the group of students who made significant progress showed a significantly lower number of posts with two functions: 1) questions (asking questions to and answering questions from teachers in the discussion forum) (F = 92.282, *df* = 2, *p* = 0.000) and 2) ideas (sharing their ideas and responses to teachers’ posts without showing their links to learning materials and other academic sources) (F = 47.675, *df* = 2, *p* = 0.000). Therefore, students who demonstrated significant progress in their reading assessments engaged in fewer peer-peer and peer-teacher interactions on the online discussion forum, contrary to previous research. This behavior is most likely due to their poor performance in the pre-test or low self-efficacy levels.

**Table 4:** Scores differences in the three groups.

	Pre-test scores		Post-test scores	
	M	SD	M	SD
Little progress (n = 84)	6.238	0.785	6.452	0.599
Moderate progress (n = 142)	5.366	0.479	6.623	0.391
Significant progress (n = 109)	4.742	0.454	6.995	0.492

**Table 5:** ANOVA test results.

	Groups	N	Mean	SD	Sig.
Social, affective, communal	1	84	12.15	5.869	0.316
	2	142	11.11	5.644	
	3	109	11.70	3.471	
Information	1	84	5.44	2.967	0.178
	2	142	4.95	2.784	
	3	109	5.54	2.279	
Question	1	84	3.08	1.530	0.000
	2	142	1.15	0.974	
	3	109	1.22	0.857	
Idea	1	84	8.56	2.872	0.000
	2	142	4.88	2.624	
	3	109	5.43	3.047	
Agency	1	84	.08	.278	0.084
	2	142	.08	.268	
	3	109	.02	.135	
Community: meta-discourse	1	84	.05	.214	0.066
	2	142	.07	.257	
	3	109	.01	.096	

To be more specific, participants in the group with little progress had an average of 3.08 posts about questions, whereas participants in the groups with moderate and significant progress only posted 1.15 and 1.22 questions, respectively. Furthermore, students in groups with little progress wrote 8.56 posts on average to express their ideas and thoughts about their teachers’ topics without citing any academic reference, whereas students in groups with moderate and significant progress contributed 4.88 and 5.43 posts in the same categories, respectively. This suggests that participants who posted more on the discussion forum to respond to the teacher’s topics and engage in peer-peer interactions by asking and answering questions showed relatively little progress between the two reading assessments.

These findings have at least two possible interpretations. One possible explanation is that these students may prefer learning from online resources in a less social setting and avoid collaborating with colleagues in an online environment. Therefore, their progress between the two reading assessments could primarily be due to their independent or offline studies. Another possible explanation is that students who made the most progress on the post-reading test had relatively low pre-test scores. Thus, asking and answering far fewer questions and sharing fewer ideas to respond to teachers’ posts could reflect a lack of confidence in communicating or a reluctance to engage with higher-achieving students. Consequently, they may have chosen to read and learn from other people’s posts instead.

Interestingly, guided by self-efficacy theory in an online learning environment, the findings from self-reflective journals of the participants in the third group lend support to the two possible explanations. In general, learner-content interactions have been reported to be more favorable and beneficial than learner-learner interactions in terms of learning and knowledge generation, sharing and communicating new knowledge with others, and self-regulating learning behaviors in an online discussion forum. In this regard, learner-instructor interactions were identified as an effective means for facilitating, regulating, and assessing the effectiveness of learner-learner and learner-content interactions.

Initial analysis reveals that peer-content interactions were identified as the predominant mode of learning in the discussion forum for the low-achieving students who demonstrated significant progress in group 3. It was reported that these students in the pre-reading assessment engaged in studying the reading materials uploaded by their teachers to the discussion forum. All of the participants who demonstrated significant progress expressed appreciation for the rich learning resources and the flexibility of studying at their own pace. However, merely 12 out of the 109 students reported engaging in peer-to-peer discussions. When encountering difficulties in engaging with the uploaded learning resources, these students desired some level of teacher involvement. For example, one student wrote in their self-reflective journal:

There are too many tasks and exercises and I can’t find those exercises which match my level… The teacher didn’t talk about the questions or give the answers. I didn’t know where he was wrong.

Furthermore, the reflective journals of the participants reveal two distinct themes. Some participants who demonstrated significant progress reported that the discussions with their peers provided new or simplified versions of the reading materials uploaded by their teachers, which helped in their comprehension process. One participant expressed this sentiment by stating:

Some others posted hot topics on the forum. I feel these news reports are not easy to read. I sometimes searched the Chinese translated version of that news and sometimes I read others’ comments below. it’s fun to read their discussions on what is happening in the world.

In addition to the provided reading and multimedia resources, the discussions and debates among high-achieving students in the discussion forum have also been recognized as valuable examples by low-proficiency students. Specifically, some students reflected that they had acquired linguistic resources from their peers’ contributions, as these peer-made comments were found to be more accessible than the original reading materials. One participant expressed this sentiment by stating:

Forum is more helpful to me, the students with higher English proficiency are more mature in the application of vocabulary and sentences, and there are many knowledge points worth learning. It is of great help to me in using them.

None of the participants in the third group reported on their experiences of exchanging and sharing ideas and responses to the teacher’s tasks and reading materials. Instead, most participants indicated that the purpose of conveying and communicating ideas and knowledge in the discussion forum was to solicit feedback on their learning outcomes and assess their understanding of the learning materials. As a result, the most common concerns or grievances regarding peer-to-peer interactions were: 1) delayed comments and feedback; 2) indirect or ambiguous responses to questions; and 3) difficulty locating relevant information in a hierarchically structured discussion forum. The majority of participants expressed their expectations for teacher feedback and evaluation of their contributions to the discussion forum. For instance, two participants wrote:

1. This is an online class and I think the interactions between teachers and us is more difficult…so the learning atmosphere is not good enough, because no immediate corrections and clarifications from the teachers are available. I want to read others’ comments on my thoughts.

2. I think the discussion on the forum can be improved and it is better to make comments and replies in the same period… because, in this way, the answers and explanations from teachers or others can be more timely. Currently, the answers and comments in the forum are in a different place. I have to search for the answers and useful explanations from a long thread of discussion. I think it is better to receive the answer or comments on my post directly, it can save me a lot of time.

Finally, it is worth noting that all students in the third group raised concerns about their self-regulating and self-directed learning strategies, particularly with regards to the materials and engaging with peers in the forum. In addition to reminding themselves to adhere to daily short-term goals, two solutions have been proposed to address these concerns. Firstly, a new function has been suggested to allow the online discussion forum to send reminders or notifications when their questions or comments receive feedback and responses from teachers and peers. This could help students feel more connected and engaged with the learning community. For example, one learner commented:

Based on the reading materials in the discussion forum, it is better for the platform to give me an English vocabulary test every night. Otherwise, I worry I may not be able to study and revise them regularly. It will be better to have answers and resolutions on the same day, so I can read these comments. Alternatively, I guess the platform can send me an email or message to remind me when new answers or new posts are left in the discussion form from the teacher or my classmates.

Second, some students suggest that teachers upload learning materials and organize a scheduled online discussion. As a result, they are not required to organize and plan their own time in order to engage with the tasks and others. For example, one learner wrote:

Downloading the videos and learning by myself is not good at all. I may recommend the teacher to organize us to watch and study it at the same time. I can learn from others while we are watching together. I can read others’ comments and discussions. It creates a sense of community.

## Discussion

To address the first research question, a statistical analysis of the quantified posts from students indicates that low-achieving students who demonstrated significant progress in the pretest left fewer posts in response to teacher posts, as well as fewer questions and answers exchanged with peers in the online discussion forum. This suggests that for low-achieving and low-self-efficacy students, learner-learner and learner-teacher interactions may be less effective or appealing than learner-content interactions in terms of improving academic performance. The relationship between high levels of engagement and improved learning outcomes in an online learning environment has been established in several studies [39, 40]. Specifically, social engagement and peer feedback have been widely recognized as two effective solutions to address high dropout rates and low motivation. For instance, Yang et al. [41] conducted a survival analysis on a MOOC dataset to investigate the social behaviors that might be associated with student dropouts on a weekly basis.

In contrast, this study argues that learner-content interactions in an online learning environment may be more efficacious in facilitating significant progress among low-achieving or low-efficacy students compared to the other two interaction types, namely learner-learner and learner-teacher interactions. This finding may also hold true for some high-achieving language learners, as highlighted in Zhang and Cui’s [25] study conducted at a prestigious university in China, which proposed that learner-teacher and learner-learner interactions are more easily accomplished in an offline rather than an online learning context.

The qualitative analysis of reflective journals from the third group provided further insights into the findings of the quantitative analysis, addressing the second research question guided by the self-efficacy literature. Low-achieving students may encounter various challenges when participating in online discussions [42]. Learner-content interactions, particularly learner-generated content, may assist them in learning and acquiring new information and linguistic knowledge from online discussion forums’ learning materials. Specifically, linguistically simplified versions of learning materials created by their peers, as well as responses and discussions between peers on tasks assigned by their teachers, were found to enhance their learning process. In other words, low-achieving students preferred engaging with learning materials created by their peers rather than their instructors. Learning from examples provided by peers was perceived as effective in preparing students for structured and organized learning, raising their motivation levels when preparing for participation in subsequent classroom activities. For instance, Bouwmeester, de Kleijn, and van Rijen [43] reported a positive correlation between students’ preparation work for seminars and the introduction of pre-seminar peer feedback sessions, such as completing study materials and comparing their answers to the tasks with peers. Therefore, those who participated in peer feedback sessions before the seminars were more likely to hold favourable attitudes and reported a higher level of engagement in plenary discussions.

Furthermore, low-achieving students in the study did not engage in peer interactions or discussions, but rather preferred to learn from their peers’ discussions and eagerly awaited feedback and suggestions on their contributions to the discussion forum. This finding can be viewed from multiple perspectives. Firstly, students with low self-efficacy may encounter more difficulties in interacting with peers and instructors in an online learning environment, as their beliefs about language learning have been linked to teacher-centered approaches in face-to-face contexts [18, 44]. For instance, Zhang et al. [18] argued that MOOC participants who engage in online discussions in large groups may not experience better learning outcomes than those who engage in synchronous paired discussions. Additionally, for students with low language proficiency, the goal of participating in online discussions may not be to share ideas but rather to seek assistance and engage in self-evaluation [45]. For example, Filius et al. [46] found that studying and evaluating the quality of peer feedback can stimulate longer dialogues among peers and constant self-reflection in small, private online courses. When feedback from peers and instructors did not align, peers were seen to challenge and question the quality of peer feedback, resulting in a deep learning process.

Lastly, the present study found that low-achieving students used peer-peer and peer-teacher interactions as a means to regulate and monitor their online learning experience, due to their perceived lack of self-discipline and time management skills. This observation is consistent with previous studies which have identified a low instructor-to-learner ratio as a challenge for both MOOC learners and instructors, as it hinders effective engagement and learning outcomes [47, 48]. To overcome this challenge, teachers need to first support students with low self-efficacy in developing their social and cognitive strategies in an online environment, before they can effectively leverage learner-learner and learner-instructor interactions. However, it should be noted that discussion forums have limitations, such as low overall participation and a lack of responsiveness [49]. To address these issues, collaboration in MOOCs needs to be supported by personalized interventions, guidance in identifying suitable peers for information exchange, and the formation of learning groups.

## Implications

This study has some important implications. At the theoretical level, unlike other studies that always emphasize the merits of peer-peer and peer-instructor interactions, this study confirms and explains the value of peer-content interactions in MOOC learning environments, especially for low achieving students. At a practical level, the present study has important implications for MOOC instructors in terms of supporting low self-efficacy learners to engage in meaningful interactions with their peers. Such learners may benefit from observing and learning from contributions made by others in a vicarious way, as well as from engaging in interactions with their peers. To meet the unique needs of low-efficacy learners, the design and administration of learning activities in MOOCs may need to be adjusted accordingly. Additionally, online discussion forums may serve as a valuable learning resource for low-efficacy MOOC learners, facilitating active engagement with online learning resources through postings, questions, answers, and discussions between peers. It is worth noting that materials co-constructed by other learners on MOOC platforms may be as academically beneficial as those provided by teachers.

## Limitation and future research avenues

The limitations of this study can be summarized into two areas: first, to answer the second question, only self-reported journals were used, which could possibly be better described through focus group interviews. Second, the limited size of our sample would lead to less accurate estimates of the underlying population. Relative to a broader and more representative sample, the results from our sample from a single university’s business department would be less generalizable. Future studies could build on these findings by examining a larger sample size across different subjects. Furthermore, the development of a valid survey instrument to measure MOOC learners’ interactive engagement strategies at scale may provide additional insights to confirm the study’s findings.

## Conclusion

This study concluded that the low-performing students from the pre-test who demonstrated significant progress later engaged in significantly fewer peer-peer and peer-teacher interactions in the MOOC discussion forum. The peer-content interactions appear to be more beneficial to these students from at least three perspectives. First of all, it helps them advance their learning and obtain new information and linguistic knowledge from the peer-made learning materials in the discussion forum. Secondly, these learners did not share and exchange ideas and answers with their peers very often. Instead, they preferred learning from others’ discussions and wished to get quick feedback and suggestions on their contributions to the discussion forum. Finally, peer-peer and peer-teacher interactions in the MOOC discussion forums were proposed as two solutions to regulate their online learning experience as they lack self-discipline and time-management skills.
